# Self-Catalyzed Hydrolysis of Nitrile-Containing RAFT Chain-Transfer Agent and Its Impact upon Polymerization Control of Methacrylic Monomers

**DOI:** 10.1021/acsmacrolett.4c00112

**Published:** 2024-04-18

**Authors:** Åsa Jerlhagen, Olivia Wilson, Eva Malmström

**Affiliations:** † KTH Royal Institute of Technology, Department of Fiber and Polymer Technology, School of Engineering Sciences in Chemistry, Biotechnology and Health, Teknikringen 56, SE-100 44 Stockholm, Sweden; ‡ FibRe − Centre for Lignocellulose-based Thermoplastics, KTH Royal Institute of Technology, Department of Fiber and Polymer Technology, School of Engineering Sciences in Chemistry, Biotechnology and Health, Teknikringen 56, SE-100 44 Stockholm, Sweden; § Wallenberg Wood Science Center, Department of Fibre and Polymer Technology, KTH Royal Institute of Technology, Teknikringen 56−58, SE-100 44 Stockholm, Sweden

## Abstract

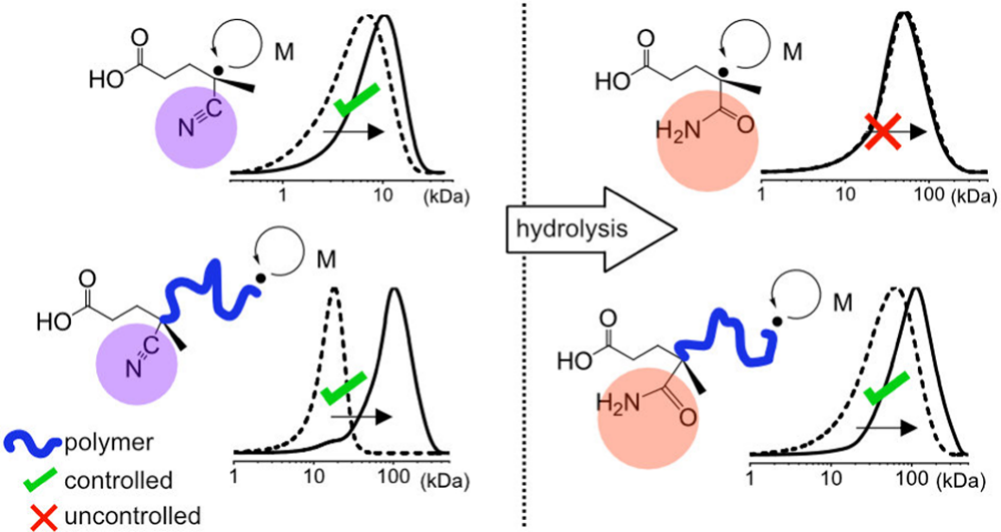

Self-catalyzed hydrolysis upon storage of the common RAFT chain-transfer agent (CTA) 4-cyano-4-[(thiothiopropyl)sulfanyl] pentanoic acid (CTPPA) is confirmed, where the nitrile group is transformed into an amide by catalysis from the adjacent carboxylic acid moiety. The amide-CTA (APP) is found to poorly control molecular weight evolution during polymerization of two methacrylates, methyl methacrylate (MMA) and *N*,*N*-(dimethylamino)ethyl methacrylate (DMAEMA), likely due to poor reinitiation speed in the pre-equilibrium. However, when attached to a macromolecule, the impact of this amide moiety becomes insignificant and chain extension proceeds as expected with CTPPA. In light of CTPPA and similarly hydrolyzable CTAs being extensively employed for aqueous polymerizations of methacrylates, these findings highlight the importance of CTA purity when performing RAFT polymerizations.

Reversible addition–fragmentation chain-transfer (RAFT) polymerization is a powerful and versatile technique for the synthesis of well-defined polymers with complex architectures.^1^ The ease of implementation of the RAFT system and its tolerance to various conditions and monomer types has quickly made it one of the most employed reversible-deactivation radical polymerization (RDRP) techniques.^2−4^ As such, its use has spread beyond the field of polymer chemistry and into more application-focused fields such as nanomedicine, theranostics, coatings, and tailored additives.^5−9^

Integral to the RAFT system is the chain transfer agent (CTA), which mediates the growing polymer chain end through degenerative transfer and allows for linear evolution of molecular weight, low dispersities (*Đ*), and the construction of complex chain architectures such as block, star, and gradient copolymers through chain extension. The CTA typically consists of a sulfur-rich organic center such as xanthate, dithioester, or trithiocarbonate with a labile carbon–sulfur double bond, allowing for fast reaction to and cleaving from the radical on the growing polymer chain end (Scheme 1a–c).^10^ The CTA’s central group is capped by the Z and R groups; where the Z group mediates the activity toward free radical addition to ensure appropriate transfer activity with the radical chain end, and the R group allows for fast reinitiation in the pre-equilibrium (Scheme 1b).^2,3^ The chain transfer coefficient (*C*_tr_) defines the efficacy of a chain transfer agent under a certain set of conditions, especially the monomer choice, and is defined according to the RAFT equilibrium constants (Scheme 1d).

**Scheme 1:**
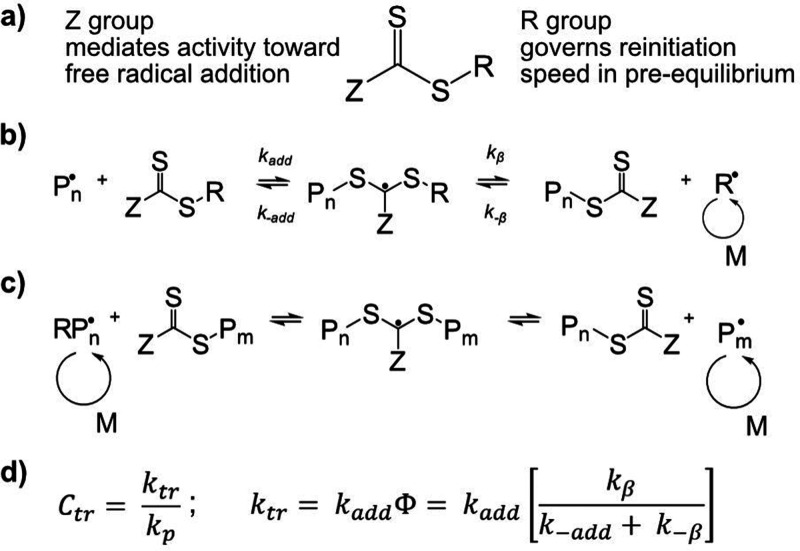
Important Steps in RAFT Polymerization (a) Structure of the chain-transfer agent CTA; (b) The RAFT pre-equilibrium; (c) Main RAFT equilibrium; (d) Definition of the chain transfer coefficient *C*_tr_.

Methacrylates are especially sensitive to the choice of Z and R group, where high activity is required to efficiently control chain growth.^10,11^ Z groups suitable for methacrylate polymerization are S-alkyl(trithiocarbonate) and phenyl(dithioester) groups, and the choice of R groups that are most suited to provide fast reinitiation are nitrile- and phenyl-containing groups.^12^ Apart from their high activity and suitability for methacrylate polymerization, trithiocarbonates have become a popular choice of CTA, because they demonstrate greater resistance to hydrolysis and attack from nucleophiles than dithioesters and, as a consequence, they have become the most commonly employed CTA class for aqueous polymerizations.^13^ One such trithiocarbonate is 4-cyano-4-[(thiothiopropyl)sulfanyl] pentanoic acid (CTPPA), which has been utilized extensively for aqueous and heterogeneous polymerizations.^14−19^ CTPPA is one of several prevalent CTAs, where the R group, 4-cyanopentanoic acid, stems from the azo-initiator 4′,4′-azobis(4-cyanopentanoic acid) (ACPA).

It is well-known that CTAs can undergo light-initiated degradation, aminolysis, cyclizations, and hydrolysis under certain conditions.^20−25^ Additionally, a few studies have shown that the 4-cyanopentanoic acid group can undergo self-catalyzed hydrolysis to yield an amino adduct.^26,27^ In one study, Fuchs et al. reported the hydrolysis of trithiocarbonates containing this R group and various Z groups (thiododecanyl, thiobutanyl, thioethanyl, and 2-phenyl(thioethanyl)) and investigated the impact on polymerization control of methyl methacrylate (MMA). Molecular weight and *Đ* increased with the addition of the amine adduct to pure CTA, indicating a loss of control. This was suggested to be caused by a lower *C*_tr_ for the amine adduct due to slow reinitiation of the R group (Figure 1b,d). DOSY-NMR showed the incorporation of the amide into the polymer chain, confirming its participation in RAFT polymerization. In the second report, CTPPA was shown to undergo the same hydrolysis upon addition of HCl, and the amino-adduct was used for polymerization of poly(ethylene glycol) methyl ether methacrylates (OEGMA and DEGMA).^27^ The amide-CTA, 4-amino-4-[(thiothiopropyl)sulfanyl] pentanoic acid (APP), produced polymers of larger molecular weights when explored under similar conditions. This was also attributed to slow reinitiation of the R group.

**Figure 1:**
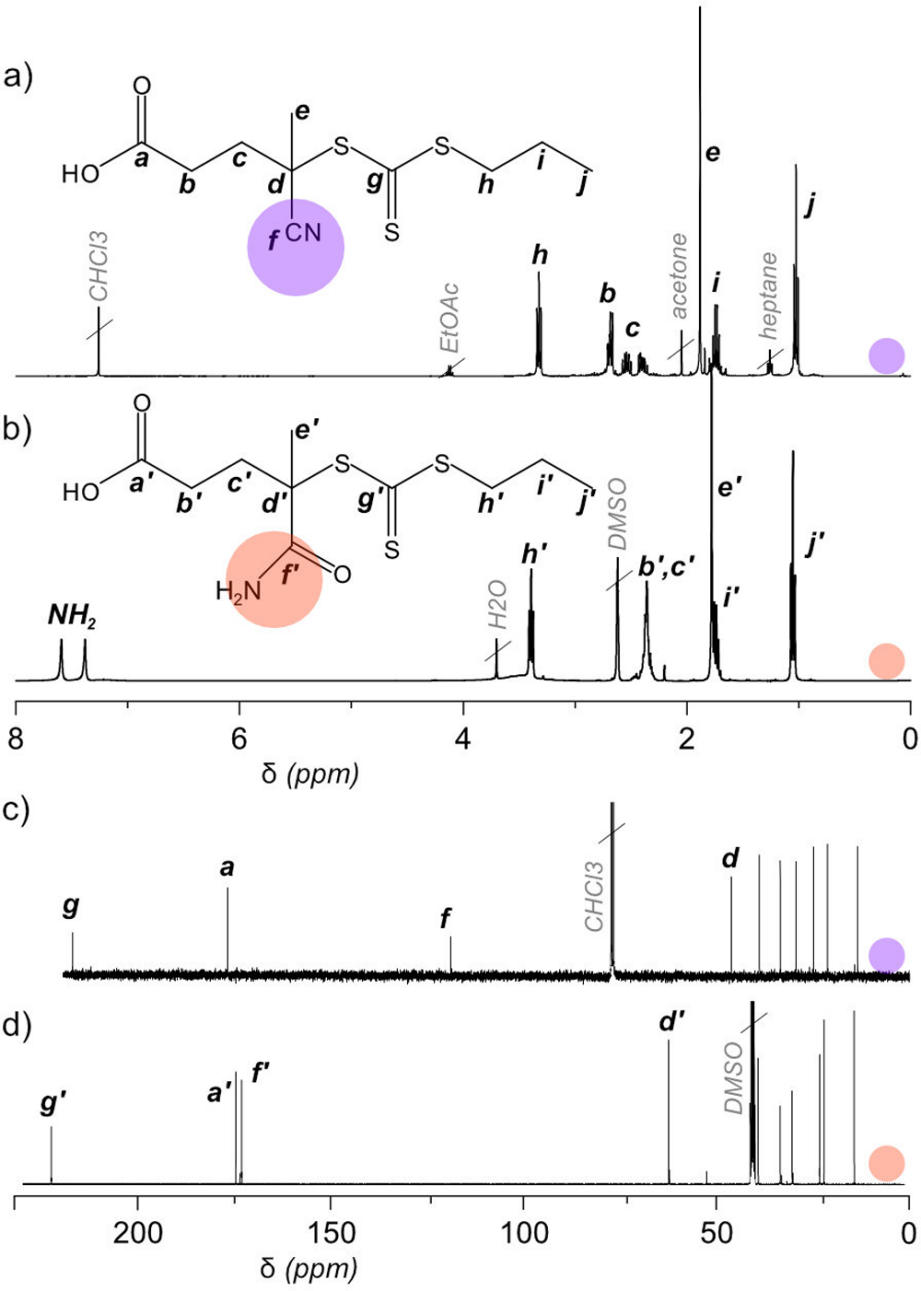
NMR characterization of nitrile-containing CTPPA (a, c) and amide-containing APP (b, d) in ^1^H NMR (a, b) and ^13^C NMR (c, d). Hydrolysis is indicated clearly by the appearance of NH_2_ signals at 7.6 and 7.4 in ^1^H NMR (b) and a shift in the nitrile peak from 119 to the carbonyl region 173 ppm in ^13^C NMR (c, d).

The previous studies illustrate the need for further exploration of the impact of CTPPA hydrolysis on polymerization control, especially with methacrylate monomers. In light of CTPPA’s importance in aqueous and heterogeneous polymerizations in particular, hydrolysis may occur not only prior to but also during polymerization and in workup and storage. In this work, we investigate the impact with two methacrylates of relevance to our work; methyl methacrylate (MMA) and *N*,*N*-(dimethylamino)ethyl methacrylate (DMAEMA).

Upon storage of CTPPA (under argon at +5 and −20 °C), it was observed that a yellow solid formed in the dark red oil. Dissolved in deuterated chloroform, faint new peaks showed up in ^1^H NMR, indicative of a degradation product. However, the solubility of this solid was seemingly low in chloroform, and a better signal was achieved in DMSO. This finding highlights that it may be difficult to determine when degradation of CTPPA has occurred, as its degradation product is only sparingly soluble in chloroform. After storage for 2 and 5 months at 5 °C, 10 and 31% of CTPPA was degraded (Figure S4). This quantification is tentative, as the resulting mixture is inhomogeneous.

To isolate the hydrolyzed product from CTPPA, the yellow solid was reprecipitated from acetone. As opposed to previous reports where this compound was synthesized by the addition of HCl,^27^ it was observed that the compound readily formed upon storage. The mechanism for self-catalyzed hydrolysis is shown in Scheme 2. It is hypothesized that the nitrile group is protonated by the adjacent carboxylic acid, allowing any water present to hydrolyze the nitrile group to an imidic acid, which tautomerizes to the amide form. The self-catalysis of the adjacent carboxylic acid is likely key to the sensitivity of this molecule and highlights the need for careful characterization of CTPPA before use, as well as an investigation of its effects upon RAFT control.

**Scheme 2:**
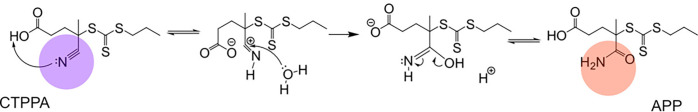
Proposed Mechanism of Hydrolysis of Chain-Transfer Agent CTPPA to Yield the Amide Form APP

The structure of APP was confirmed with NMR and FTIR. In ^1^H NMR, slight upfield shifts of methylenes at h, b, and c (from 3.32, 2.69, and 2.54–2.41 to 3.39 and 2.36), as well as singlet e (from 1.89 to 1.78), are observed (Figure 1a). To further support the presence of APP, characteristic amide signals are observed in new peaks at 7.6 and 7.4 ppm. These signals decrease in intensity upon addition of D_2_O, confirming labile protons that exchange with deuterium. ^13^C NMR shows the complete disappearance of signal f (119 ppm) as it is shifted to the carbonyl region (173 ppm). The full coupling scheme for these two molecules can be found in Figures S1 and S2. FTIR also corroborates the amide formation, as the nitrile signal at 2230 cm^–1^ disappears while amide double signals at 3320–3200 cm^–1^ (N—H stretch) and 1635–1575 cm^–1^ (C=O stretch) appear (Figure S3).

Initially CTPPA and APP were assessed as CTAs for the polymerization of MMA in THF at 70 °C. The reactions are abbreviated MMA_CTPPA, MMA_APP, and MMA_FRP for polymerization of MMA with CTPPA, APP, and without chain-transfer agent (free-radical polymerization (FRP)), respectively. The [M]/[CTA] ratio was kept constant at 770 and the [CTA]/[I] ratio at 10. Figure 2a shows a linear growth of molecular weight as a function of monomer conversion, indicating good and characteristic RAFT control. On the other hand, polymerization using APP showed molecular weight development similar to that in a free radical polymerization (Figure 2b,c), producing large molecular weights at low conversion and no increase in molecular weight during monomer conversion. Curiously, the *Đ* values of both free radical polymerizations (no CTA present) and with APP as CTA have similar values (1.3–2). The monomer conversion over time is not impacted by the choice of CTA, and no difference in inhibition period is seen (Figure S5). The similarities between MMA_APP and MMA_FRP results indicated that APP was inactive as a chain-transfer agent.

**Figure 2:**
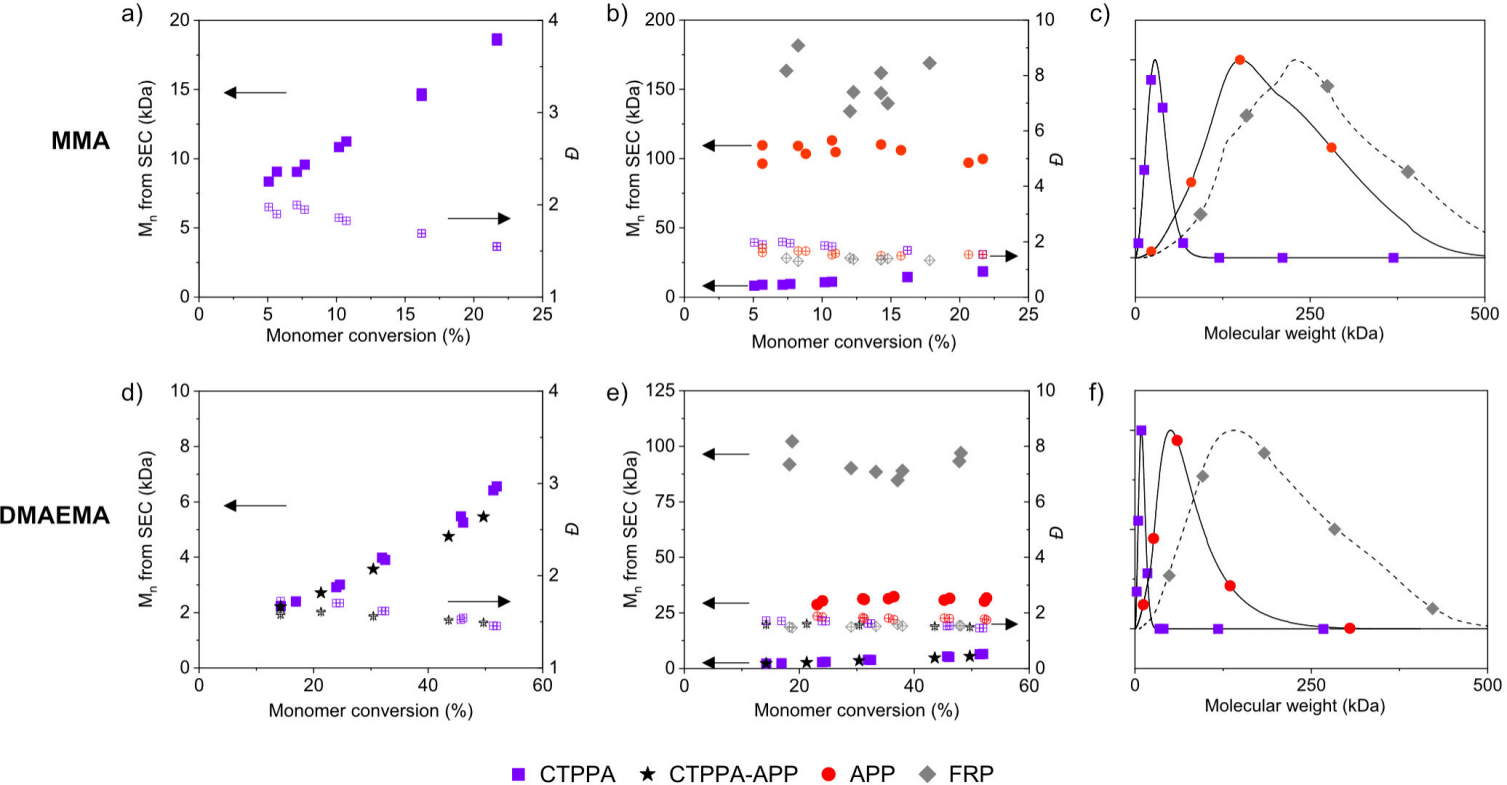
Polymerization kinetics of MMA (a–c) with CTPPA, APP, and without chain-transfer agent (free-radical polymerization, FRP), respectively. Filled symbols indicate *M*_n_ and open symbols indicate *Đ*. Elugrams (c) show differences in molecular weight distributions after 180 min of reaction (conversion ∼ 15%). Polymerization kinetics of DMAEMA (d–f) with CTPPA, APP, and without chain-transfer agent (FRP), respectively. Filled symbols indicate *M*_n_ and open symbols *Đ*. Elugrams (f) show differences in molecular weight distributions after 180 min of reaction (conversion ∼ 45%). Monomer conversion is calculated from ^1^H NMR.

CTPPA and APP were also assessed for the polymerization of DMAEMA in dioxane at 70 °C (abbreviated DMA_CTPPA, DMA_APP, and DMA_FRP). The [M]/[CTA] ratio was kept at 100, and the [CTA]/[I] ratio was kept at 10. The [M]/[CTA] ratios for these reactions were chosen to mimic systems of importance for our work, where short polymers of DMAEMA are extended with other methacrylates using polymerization-induced self-assembly (PISA). The molecular weight evolution of DMAEMA with CTPPA exhibits RAFT characteristics including linear evolution of *M*_n_ with monomer conversion and moderately low *Đ* (Figure 2d). As previous results had indicated that APP was inactive as chain-transfer agent, a 1:1 mol ratio of APP was added to the CTPPA system, doubling the total CTA concentration (DMA_CTPPA- APP). As seen for DMA_CTPPA-APP in Figure 2d, the molecular weight evolution and *Đ* of this system was unchanged. This again indicated that APP did not participate as the chain-transfer agent. However, when DMAEMA was polymerized with only APP present the molecular weights achieved were significantly lower than those found in free radical polymerization (DMA_APP versus DMA_FRP in Figure 2e,f). This was surprising, as all previous results had indicated that APP was inactive as CTA. In DMA_APP, the molecular weights were significantly lower than those in FRP, but they did not increase with conversion. In all DMAEMA polymerizations, the consumption of monomer with time and *Đ* were not impacted by the choice of CTA (Figure S7). This strange molecular weight evolution in the DMA_APP polymerizations indicated that APP was indeed active as chain transfer agent and led us to look closer at different approaches to quantify its transfer coefficient *C*_tr_.

The transfer constant, *C*_tr_ as defined in Scheme 1d, describes the activity of a certain CTA in a RAFT equilibrium. From the experimental results, the apparent transfer coefficient *C*_tr,app_ can be approximated using different strategies. In previous reports, *C*_tr,app_ of similar amide-functional CTAs with different Z groups have been approximated using differences in *Đ*, as derived elsewhere.^10^ Fuchs et al. found that *C*_tr,app_ decreased drastically with a factor of about 5 for CTAs with the amide moiety.^26^ As the dispersity is not greatly impacted in this work, calculating the *C*_tr,app_ using this method yields insignificant differences.

Another strategy for approximating CTA activity is based upon the differences between theoretical molecular weight as calculated from conversion values (*X*_*n*_(calc)), with the measured molecular weight from size exclusion chromatography (*X*_*n*_(found)). This method yields an estimation of the CTA conversion during the course of the reaction according to eq 1, as derived fully by Chiefari et al.^11^*C*_tr,app_ can then be calculated according to eq 2 by the slope of the plot ln([CTA]_inst_) and ln([M]_inst_), where [CTA]_inst_ and [M]_inst_ are the instantaneous molar concentrations of CTA and monomer respectively as calculated from monomer and CTA conversion (eq 2).
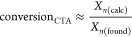
1
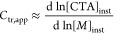
2

The values for CTA conversion in Figure 3a show that the conversion of APP in MMA polymerization was low but that APP is indeed not inactive. Using eq 2, we approximate *C*_tr,app_ to 9.2 and 0.8 for CTPPA and APP, respectively (Figure 3b). These values can be calculated for PMMA polymers as the SEC columns are calibrated using PMMA standards. For the DMAEMA system, CTA conversion is approximated according to eq 1 (Figure 3c), but because the molecular weights from SEC are relative to PMMA standards, the conversion values can only be compared, and *C*_tr,app_ cannot be calculated. Qualitatively, however, we can see that the reason for poor control using APP is due to the low conversion of APP during the course of the reaction. The low CTA conversion in these reactions gives rise to larger molecular weights than predicted, as the instantaneous [M]/[CTA] is higher than that targeted. For both MMA and DMAEMA polymerization, we can thus conclude that APP is active, but that its conversion is low.

**Figure 3:**
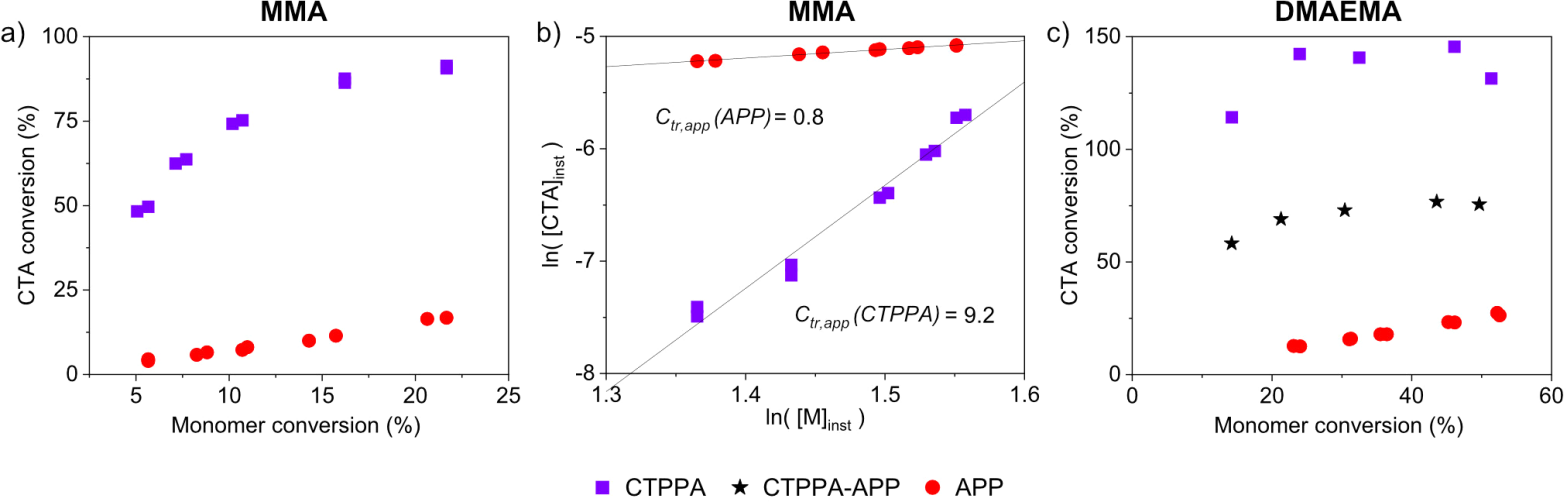
Conversion of CTA is calculated from the difference between the found chain length *X*_*n*_(found) (as calculated from SEC) and expected chain length *X*_*n*_(calc) (as calculated from ^1^H NMR) for MMA polymerization (a) and DMAEMA polymerization (c). Monomer conversion is calculated from ^1^H NMR. The apparent transfer coefficient *C*_tr,app_ is approximated for MMA polymerizations according to eq 2, showing a 10-fold decrease in transfer coefficient between CTPPA and APP.

The sensitivity of methacrylates to differences in R group has been studied before, most notably in early RAFT papers.^12^ Here it was shown that only tertiary cyanoalkyl and cumyl R groups were sufficiently fast in reinitiating MMA polymerizations, whereas alkyl and ester R groups appeared ineffective. In this regard, it is not surprising that the hydrolysis of CTPPA to APP renders CTA unsuccessful in methacrylate polymerization.

Apart from linear molecular weight evolution, another important feature of RAFT polymerization is the active chain end, which can be extended by further monomer conversion to access well-defined and advanced polymer architectures. Whether the amide functionality is incorporated from APP or from hydrolysis of the CTPPA’s nitrile group during polymer workup and storage, it is of vital importance that the polymer still retains active chain ends for building complex architectures through chain extension. It has been shown previously by DOSY NMR that amide-functional CTAs are incorporated into the chain.^26^ As we were unable to resolve the end-group of APP using NMR in this work, its incorporation could not be corroborated. Instead, we chose to investigate whether polymers produced with APP retain the functionality of chain extendibility.

A chain extension experiment was designed to investigate whether APP-functional PDMAEMA can be chain extended and whether the presence of APP impacts the extendability of chain ends. [M]/[CTA] was kept at 1000 and the reactions were performed at 70 °C. Figure 4b shows the chromatograms of the extension of PDMAEMA with CTPPA (CE_CTPPA) and with APP (CE_APP). Both of these systems are extendable, corroborating that APP alone can act as a chain-transfer agent (Figure 4). In Figure 4a, the chain growths appear to be similar. These results indicate that the macroCTA with the APP R group (4-amino-propanoic acid) is as efficient a macroCTA as with the CTPPA R group (4-cyano-propanoic acid). The PDMAEMA polymer attached to the trithiocarbonate Z group now acts as the R group and the poor activity of the amide becomes insignificant. Therefore, when the R group is a sufficiently large polymer of, e.g., PDMAEMA, the hydrolysis of the nitrile does not impact chain extendibility.

**Figure 4:**
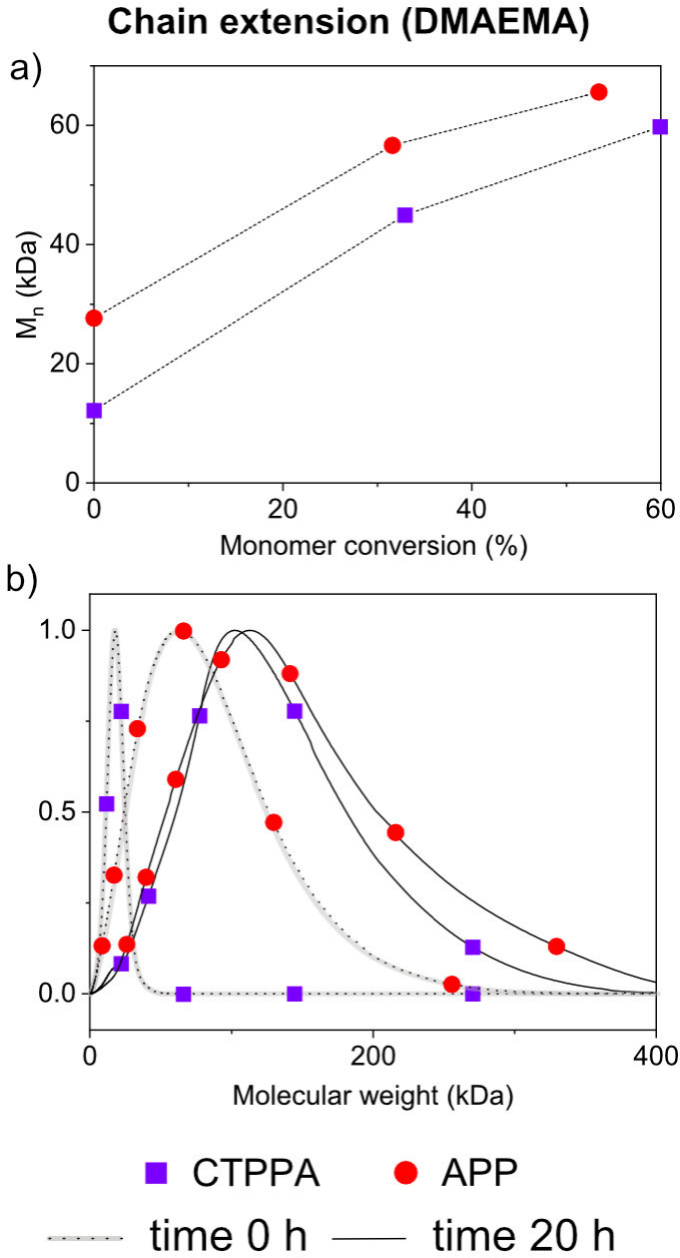
Chain extension experiments were performed with PDMAEMA macroCTAs made with CTPPA and APP. *M*_n_ with monomer conversion (a) shows similar chain extension behavior, and elugrams (b) show growth of polymers from time 0 (conversion = 0) and after 20 h (conversion 55–60%).

In this study, hydrolysis under storage of the popular chain-transfer agent CTPPA is confirmed. The R group contains a nitrile that is adjacent to a carboxylic acid, which readily hydrolyzes to its amide form APP through self-catalysis. It has been found that APP alone is as active as CTA but has poor chain-transfer activity in polymerizations of two methacrylates (MMA and DMAEMA), leading to unpredictable molecular weight evolution. If a small amount of hydrolysis has occurred in CTPPA upon storage, the user is likely to see molecular weights that are larger than expected. However, chain extension experiments show that when attached to a sufficiently large polymer, the presence of the amide group does not impact chain growth. This means that a polymeric CTA synthesized with CTPPA can be trusted to extend the chain as predicted, even though nitrile hydrolysis has occurred at the chain end. The current results highlight the importance of CTA purity when performing RAFT polymerizations and constitute a word of caution when using any of the popular commercial and noncommercial CTAs containing the 4-cyano-4-methylpentanoic acid as the R group.

## Supporting Information Available

Experimental Section; Table S1; Figures S1–S10 (PDF)

## References

[ref1] Chiefari J.; Chong Y. K.; Ercole F.; Krstina J.; Jeffery J.; Le T. P. T.; Mayadunne R. T. A.; Meijs G. F.; Moad C. L.; Moad G.; et al. Living Free-Radical Polymerization by Reversible Addition–Fragmentation Chain Transfer: The RAFT Process. Macromolecules 1998, 31 (16), 5559–5562. 10.1021/ma9804951.

[ref2] Perrier S. 50th Anniversary Perspective: RAFT Polymerization—A User Guide. Macromolecules 2017, 50 (19), 7433–7447. 10.1021/acs.macromol.7b00767.

[ref3] Keddie D. J. A guide to the synthesis of block copolymers using reversible-addition fragmentation chain transfer (RAFT) polymerization. Chem. Soc. Rev. 2014, 43 (2), 496–505. 10.1039/C3CS60290G. 24129793

[ref4] Postma A.; Skidmore M. How to Do a RAFT Polymerization. In RAFT Polymerization, Methods, Synthesis and Applications; Moad G., Rizzardo E., Eds.; John Wiley and Sons, 2021; Vol. 2, pp 25–58.

[ref5] Crucho C. I. C. Stimuli-Responsive Polymeric Nanoparticles for Nanomedicine. ChemMedChem. 2015, 10 (1), 24–38. 10.1002/cmdc.201402290. 25319803

[ref6] Pearce A. K.; Rolfe B. E.; Russell P. J.; Tse B. W. C.; Whittaker A. K.; Fuchs A. V.; Thurecht K. J. Development of a polymer theranostic for prostate cancer. Polym. Chem. 2014, 5 (24), 6932–6942. 10.1039/C4PY00999A.

[ref7] Li B.; Li X.; Zhang K.; Li H.; Zhao Y.; Ren L.; Yuan X. Synthesis of POSS-containing fluorosilicone block copolymers via RAFT polymerization for application as non-wetting coating materials. Prog. Org. Coat. 2015, 78, 188–199. 10.1016/j.porgcoat.2014.09.004.

[ref8] Moad G.; Chen M.; Häussler M.; Postma A.; Rizzardo E.; Thang S. H. Functional polymers for optoelectronic applications by RAFT polymerization. Polym. Chem. 2011, 2 (3), 492–519. 10.1039/C0PY00179A.

[ref9] Semsarilar M.; Abetz V. Polymerizations by RAFT: Developments of the Technique and Its Application in the Synthesis of Tailored (Co)polymers. Macromol. Chem. Phys. 2021, 222 (1), 2000311 10.1002/macp.202000311.

[ref10] Buback M. Kinetics and Mechanism of RAFT Polymerizations. In RAFT Polymerization, Methods, Synthesis and Applications; Moad G., Rizzardo E., Eds.; John Wiley and Sons, 2021, Vol. 2, pp 59–93.

[ref11] Chiefari J.; Mayadunne R. T. A.; Moad C. L.; Moad G.; Rizzardo E.; Postma A.; Skidmore M. A.; Thang S. H. Thiocarbonylthio Compounds (SC(Z)S–R) in Free Radical Polymerization with Reversible Addition-Fragmentation Chain Transfer (RAFT Polymerization). Effect of the Activating Group Z. Macromolecules 2003, 36 (7), 2273–2283. 10.1021/ma020883+.

[ref12] Chong Y. K.; Krstina J.; Le T. P. T.; Moad G.; Postma A.; Rizzardo E.; Thang S. H. Thiocarbonylthio Compounds [SC(Ph)S–R] in Free Radical Polymerization with Reversible Addition-Fragmentation Chain Transfer (RAFT Polymerization). Role of the Free-Radical Leaving Group (R). Macromolecules 2003, 36 (7), 2256–2272. 10.1021/ma020882h.

[ref13] Moad G. Trithiocarbonates in RAFT Polymerization. In RAFT Polymerization, Methods, Synthesis and Applications; Moad G., Rizzardo E., Eds.; John Wiley and Sons, 2021, Vol. 2, pp 359–492.

[ref14] Chaduc I.; Crepet A.; Boyron O.; Charleux B.; D’Agosto F.; Lansalot M. Effect of the pH on the RAFT Polymerization of Acrylic Acid in Water. Application to the Synthesis of Poly(acrylic acid)-Stabilized Polystyrene Particles by RAFT Emulsion Polymerization. Macromolecules 2013, 46 (15), 6013–6023. 10.1021/ma401070k.

[ref15] Carlsson L.; Fall A.; Chaduc I.; Wågberg L.; Charleux B.; Malmström E.; D’Agosto F.; Lansalot M.; Carlmark A. Modification of cellulose model surfaces by cationic polymer latexes prepared by RAFT-mediated surfactant-free emulsion polymerization. Polym. Chem. 2014, 5 (20), 6076–6086. 10.1039/C4PY00675E.

[ref16] Pavlovic M.; Adok-Sipiczki M.; Nardin C.; Pearson S.; Bourgeat-Lami E.; Prevot V.; Szilagyi I. Effect of MacroRAFT Copolymer Adsorption on the Colloidal Stability of Layered Double Hydroxide Nanoparticles. Langmuir 2015, 31 (46), 12609–12617. 10.1021/acs.langmuir.5b03372. 26528779

[ref17] Bourgeat-Lami E.; França A. J. P. G.; Chaparro T. C.; Silva R. D.; Dugas P. Y.; Alves G. M.; Santos A. M. Synthesis of Polymer/Silica Hybrid Latexes by Surfactant-Free RAFT-Mediated Emulsion Polymerization. Macromolecules 2016, 49 (12), 4431–4440. 10.1021/acs.macromol.6b00737.

[ref18] Velasquez E.; Rieger J.; Stoffelbach F.; D’Agosto F.; Lansalot M.; Dufils P.-E.; Vinas J. Surfactant-free poly(vinylidene chloride) latexes via one-pot RAFT-mediated aqueous polymerization. Polymer 2016, 106, 275–284. 10.1016/j.polymer.2016.08.083.

[ref19] Engström J.; Hatton F. L.; Wågberg L.; D’Agosto F.; Lansalot M.; Malmström E.; Carlmark A. Soft and rigid core latex nanoparticles prepared by RAFT-mediated surfactant-free emulsion polymerization for cellulose modification – a comparative study. Polym. Chem. 2017, 8 (6), 1061–1073. 10.1039/C6PY01904H.

[ref20] Li S.; Han G.; Zhang W. Photoregulated reversible addition–fragmentation chain transfer (RAFT) polymerization. Polym. Chem. 2020, 11 (11), 1830–1844. 10.1039/D0PY00054J.

[ref21] Qiu X.-P.; Winnik F. M. Facile and Efficient One-Pot Transformation of RAFT Polymer End Groups via a Mild Aminolysis/Michael Addition Sequence. Macromol. Rapid Commun. 2006, 27 (19), 1648–1653. 10.1002/marc.200600436.

[ref22] Liu Y.; Cavicchi K. A. Reversible Addition Fragmentation Chain Transfer (RAFT) Polymerization with a Polymeric RAFT Agent Containing Multiple Trithiocarbonate Groups. Macromol. Chem. Phys. 2009, 210 (19), 1647–1653. 10.1002/macp.200900201.

[ref23] Rasheed K.; Warkentin J. D. Cyclization of dinitrophenyl tert-butyl trithiocarbonates. A novel synthesis of nitro-1,3-benzodithiole-2-thiones. The Journal of Organic Chemistry 1980, 45 (20), 4041–4044. 10.1021/jo01308a020.

[ref24] Ito H.; Oto K.; Takasu A.; Higuchi M. Facile synthesis of cyclic RAFT agents and ring expansion radical polymerization of vinyl monomers having cyclic topology. Polym. Chem. 2024, 15, 754 10.1039/D4PY00031E.

[ref25] Loiseau J.; Doërr N.; Suau J. M.; Egraz J. B.; Llauro M. F.; Ladavière C.; Claverie J. Synthesis and Characterization of Poly(acrylic acid) Produced by RAFT Polymerization. Application as a Very Efficient Dispersant of CaCO_3_, Kaolin, and TiO_2_. Macromolecules 2003, 36 (9), 3066–3077. 10.1021/ma0256744.

[ref26] Fuchs A. V.; Thurecht K. J. Stability of Trithiocarbonate RAFT Agents Containing Both a Cyano and a Carboxylic Acid Functional Group. ACS Macro Lett. 2017, 6 (3), 287–291. 10.1021/acsmacrolett.7b00100. 35650904

[ref27] Montoya-Villegas K. A.; Licea-Claveríe Á.; Zapata-González I.; Gómez E.; Ramírez-Jiménez A. The effect in the RAFT polymerization of two oligo(ethylene glycol) methacrylates when the CTA 4-cyano-4-(propylthiocarbonothioylthio) pentanoic acid is auto-hydrolyzed to its corresponding amide. Journal of Polymer Research 2019, 26 (3), 71 10.1007/s10965-019-1718-4.

