# Supplementary material for: Self-Catalyzed Hydrolysis of Nitrile-Containing RAFT Chain-Transfer Agent and Its Impact upon Polymerization Control of Methacrylic Monomers
Source: ACS Macro Lett. 2024 Apr 18;13(5):565–70. doi: 10.1021/acsmacrolett.4c00112 (PMC11112727; doi:10.1021/acsmacrolett.4c00112)
Supplement: Supplementary file 1 — mz4c00112_si_001.pdf [file mz4c00112_si_001.pdf]

Supporting Information

Self-catalyzed hydrolysis of nitrile-containing RAFT chain-transfer agent and its impact upon polymerization control of methacrylic monomers

Åsa Jerlhagen[a,b], Olivia Wilson[a,b,c], and Eva Malmström[a,b,c]*

[a]KTH Royal Institute of Technology, Department of Fiber and Polymer Technology, School of Engineering Sciences in Chemistry, Biotechnology and Health, Teknikringen 56, SE-100 44 Stockholm, Sweden

[b]FibRe − Centre for Lignocellulose-based Thermoplastics, KTH Royal Institute of Technology, Department of Fiber and Polymer Technology, School of Engineering Sciences in Chemistry, Biotechnology and Health, Teknikringen 56, SE-100 44 Stockholm, Sweden

[c]Wallenberg Wood Science Center, Department of Fibre and Polymer Technology, KTH Royal Institute of Technology, Teknikringen 56-58, SE-100 44 Stockholm, Sweden

Tel: +46 8 790 72 25, e-mail: mavem@kth.se

* Corresponding author

Contents

Experimental Section

Materials and Methods

Table S1. Overview Polymerizations

Supporting Data

Figure S1: NMR characterization of CTPPA.
Figure S2: NMR characterization of APP.
Figure S3: FTIR of CTPPA and APP.
Figure S4: Degradation of CTPPA to APP upon storage.
Figure S5: Conversion kinetics of polymerizations of MMA in THF.
Figure S6: Elugrams showing chain growth in polymerizations of MMA in THF.
Figure S7: Conversion kinetics of polymerizations of DMAEMA in dioxane.
Figure S8: Elugrams showing chain growth in polymerizations of DMAEMA in dioxane.
Figure S9: Elugrams showing chain growth in polymerizations of DMAEMA in dioxane.
Figure S10: Elugrams showing chain extension experiments.

Experimental Section

Materials

1-propanethiol (99 %), potassium hexacyanoferrate(II) (≥99 %), carbon disulfide anhydrous (CS_2) (≥99 %), and 4.4’-azobis(4-cyanovaleric acid) (ACVA)(98 %) were purchased from Sigma Aldrich and

used as received. Sodium hydroxide (NaOH) was purchased from VWR, and magnesium sulfate ($MgSO_4$) high purity was purchased from Acros Organics. Acetone and ethyl ether (Et_2O) were purchased from VWR, ethyl acetate (EtOAc) and heptane from Merck.

N,N-dimethylamino ethyl methacrylate (DMAEMA) (98 %) containing 700-1000 ppm monomethyl ether hydroquinone (MEHQ), and methyl methacrylate (MMA) (99 %) containing ≤30 ppm MEHQ were purchased from Sigma Aldrich, and the inhibitor was removed prior to polymerization by passing over basic alumina. Basic alumina (aluminium oxide 90 active basic (0.063-0.200 mm) activity stage I) was purchased from Merck Millipore. Tetrahydrofuran (THF) anhydrous (99 %) and dioxane anhydrous (99 %) were purchased from Sigma Aldrich and used as received.

Synthesis of 4-cyano-4-methyl((pentasulfanylthiocarbonyl)sulfanyl)pentanoic acid (CTPPA)

1.87 g NaOH (0.87 eq, 46.8 mmol) was dissolved in 2.5 ml deionized water, and added to a solution of 5.0 ml 1-propanethiol (1 eq, 55.6 mmol) in 40 ml acetone at 0°C, giving precipitation of white solid. 6.0 ml CS_2 (1.8 eq, 100 mmol) was then added to the suspension, which was left stirring for 10 minutes until homogeneous. A solution of 27.46 g potassium hexacyanoferrate(II) (1.5 eq, 83.4) mmol) in 80 ml water was added drop-wise forming yellow solids in a red solution. The suspension was left to stir for 1 hour at ambient temperature. This was extracted 2 times with Et_2O as a bright red organic phase, which was then washed with NaOH 1M to remove thiol, and washed with deionized water. The organic phase was dried with $MgSO_4$ and dried under reduced pressure. Yield 5.94 g (71 %) of red oil (bis(propylsulfanylthiocarbonyl) disulfide). ^{1}H NMR (400 MHz, CDCl3) δ 3.29 (t, J = 7.3 Hz, 2H), 1.82-1.67 (m, 2H), 1.03 (d, J = 7.3 Hz, 3H). ^{13}C NMR (101 MHz, CDCl3) δ 40.24, 21.12, 13.64.

The red oil (5.94 g, 1 eq, 19.68 mmol) was dissolved in 50 ml ethyl acetate. 6.62 g ACVA (1.2 eq, 23.62 mmol) was added and the mixture was degassed by Argon bubbling, and then refluxed at 95°C overnight under inert atmosphere. The solvent was removed and the crude was purified by silica gel column chromatography with eluent heptane:ethyl acetate (1:2). 49 % yield of red-orange oil.

FTIR-ATR (cm^{-1}): 3500-2500 (COOH), 2960-2870 (CH_2, CH_3), 2230 (CN), 1710 (C=O), 1070 (C=S), 800 (C-S). ^{1}H NMR (400 MHz, CDCl3) δ 3.32 (t, J = 7.3 Hz, 2H), 2.69 (m, 2H), 2.54-2.41 (m, 2H), 1.89 (s, 3H), 1.75 (m, 2H), 1.03 (t, J = 7.4 Hz, 3H). ^{13}C NMR (101 MHz, CDCl3) δ 216.96 (CS_3), 176.93 (COOH), 119.03 (CN), 46.34, 38.99, 33.61, 29.58, 24.99, 21.41, 13.61.

Purification of 5-amino-4-methyl-4-(propylthiocarbonothioylthio)-5-oxopentanoic acid (APP)

APP was extracted from CTPPA with visible yellow solid present through reprecipitation from acetone. Reprecipitations were repeated until TLC showed no spot from CTPPA.

FTIR- ATR (cm^{-1}): 3450 (COOH), 3320-3200 (NH_2), 2930-2700 (CH_2, CH_3), 1690 (C=O), 1635 (NH_2), 1575 (NH_2), 1060 (C=S), 800 C-S). ^{1}H NMR (400 MHz, DMSO) δ 7.59 (s, 1H), 7.38 (s, 1H), 3.39 (t, J = 7.2 Hz, 2H), 2.36 (m, 4H), 1.78 (s, 3H), 1.75 (m, 2H), 1.05 (t, J = 7.3 Hz, 3H). ^{13}C NMR (101 MHz, DMSO) δ 222.58 (CS_3), 174.77 (COOH), 173.25 ($CONH_2$), 62.49, 39.31, 33.65, 30.51, 23.42, 22.25, 14.44.

Polymerization of MMA in THF

To a 5 ml pressure vial was added CTPPA (9 mg, 1 eq, 0.032 mmol), MMA (2.66 ml, 770 eq, 25.0 mmol), ACVA (0.91 mg, 0.1 eq, 0.0032 mmol) and 2.07 ml THF. [M]/[CTA] = 770, [CTA]/[I] = 10, [M] = 5.0 mol L^{-1}. The vial was closed, cooled to -36°C, degassed with Argon, and then warmed to room temperature 30 min. Reactions were performed in 70°C oil bath. Kinetic aliquots were taken during

the course of the reaction for NMR (100 ul sample in 600 ul $CDCl_3$) and SEC (100 ul sample in 1 ml eluent).

Polymerization of DMAEMA in dioxane

To a 5 ml pressure vial was added CTPPA (34.7 mg, 1 eq, 0.125 mmol), DMAEMA (2.11 ml, 100 eq, 12.5 mmol), ACVA (3.50 mg, 0.1 eq, 0.0125 mmol) and 1.87 ml dioxane. [M]/[CTA] = 100, [CTA]/[I] = 10, [M] = 2.5 mol L^{-1}. The vial was closed, cooled to 0^oC, degassed with Argon, and then warmed to room temperature 30 min. Reactions were performed in 70 ^oC oil bath. Kinetic aliquots were taken during the course of the reaction for NMR (100 ul sample in 600 ul $CDCl_3$) and SEC (100 ul sample in 1 ml eluent). Polymers used for chain extension were precipitated once in cold heptane.

Chain extension of PDMAEMA

A macroCTA was synthesized with CTPPA or APP according to the protocol above, and reacted for 19 hours (conversion ~90 %). The polymer was precipitated in cold heptane, decanted and dried under reduced pressure to a brittle solid.

To a pressure vial was added macroCTA (109 mg, 1 eq, 0.0075 mmol), DMAEMA (1.26 ml, 1000 eq, 7.5 mmol), ACVA (0.21 mg, 0.1 eq, 0.75 µmol) and 1.74 ml dioxane. The vial was closed, cooled to 0^oC, degassed with Argon, and then warmed to room temperature for 30 min. Reactions were performed in 70^oC oil bath. Kinetic aliquots were taken at 3 and 20 h of reaction.

Characterization

NMR spectra were recorded using a Bruker Advance 400 spectrometer equipped with a 5 mm broad-band multinuclear (PABBO) probe at 25^oC. Data analysis was preformed using MestReNova software. ^{1}H-NMR was used to calculate monomer conversion by comparing intensity of the alkene signals of the monomers (5.5 – 6 ppm) to methyl signals from the polymer (0.8-1.2 ppm).

FTIR spectra were recorded with a Perkin-Elmer Spectrum 2000 FTIR equipped with a MKII Golden Gate, single reflection ATR crystal with a MKII heated diamond 45° ATR top plate (from Specac Ltd, London, UK).

Size exclusion chromatography (SEC) was performed on a TOSOH EcoSEC HLC-8320GPC system equipped with EcoSEC RI detector and three columns PSS PFG 5 µm; Microguard, 100 Å, and 300 Å; MW resolving range: 100–300 000 g mol−1) from PSS GmbH, using DMF as solvent with 0.01 M LiBr as the mobile phase at 50 °C with a flow rate of 0.2 mL min−1. PMMA standards between 700 – 2 000 000 g mol^{-1} were used for the calibration.

Overview polymerizations

Code	Code	Monomer	[M] mol/L	CTA
R1	MMA_CTPPA	MMA	5	CTPPA
R2	MMA_CTPPA	MMA	5	CTPPA
R3	MMA_APP	MMA	5	APP
R4	MMA_APP	MMA	5	APP
R5	MMA_FRP	MMA	5	-
R6	MMA_FRP	MMA	5	-
R7	DMA_CTPPA	DMAEMA	2.5	CTPPA
R8	DMA_CTPPA	DMAEMA	2.5	CTPPA
R9	DMA_APP	DMAEMA	2.5	APP
R10	DMA_APP	DMAEMA	2.5	APP
R11	DMA_FRP	DMAEMA	2.5	-
R12	DMA_FRP	DMAEMA	2.5	-
R13	DMA_CTPPA-APP	DMAEMA	2.5	CTPPA/APP 1:1
R14	CE_CTPPA	DMAEMA	2.5	R7 (CTPPA)
R15	CE_APP	DMAEMA	2.5	R9 (APP)

Table S1: Overview of polymerization reactions performed with MMA (R1-R6), DMAEMA (R7-R13) and chain extensions of PDMAEMA with DMAEMA (R14-16)

Supporting Data

Characterization of CTPPA and APP

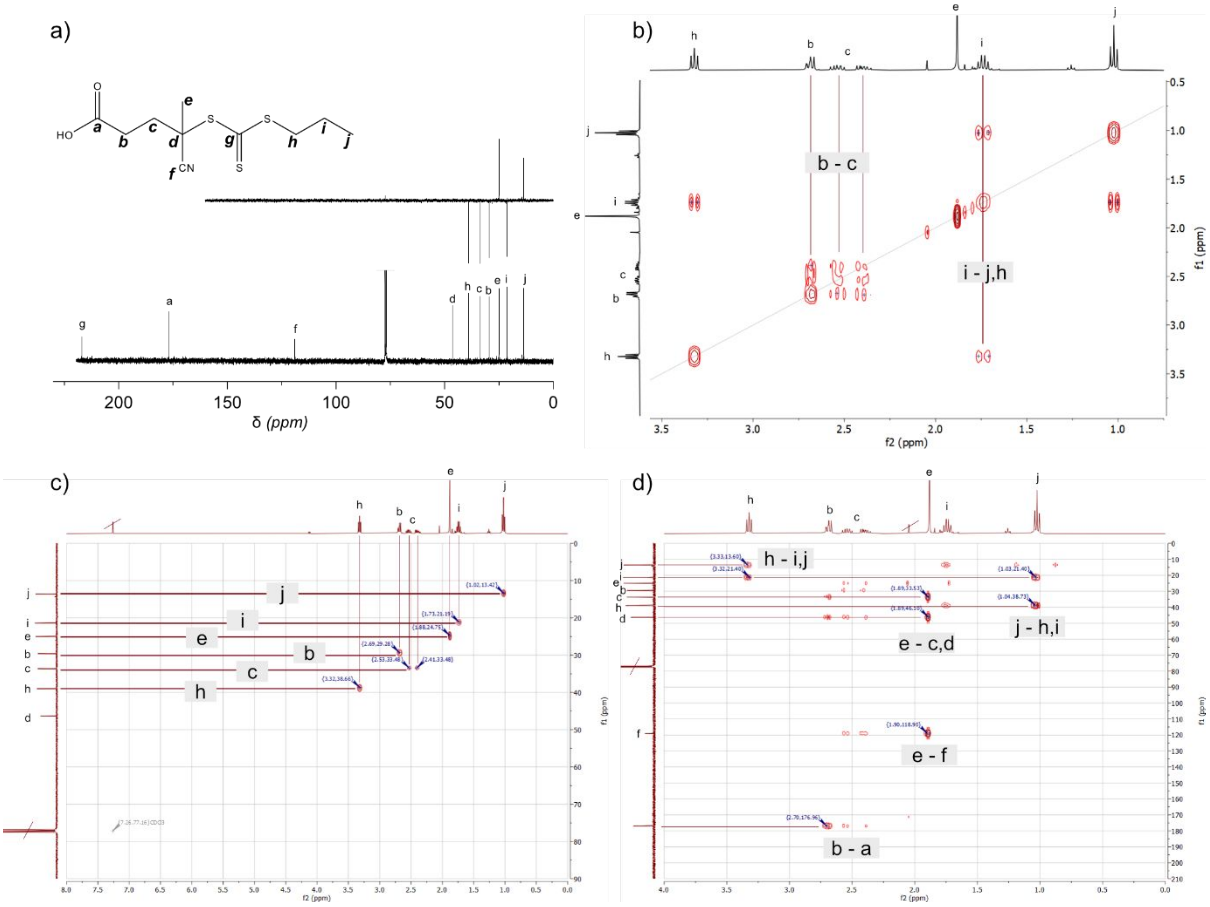

Figure S1: NMR characterization of CTPPA in deuterated chloroform; (a) ^{13}CNMR and DEPT-135, (b) COSY, (c) HSQC, and (d) HMBC.

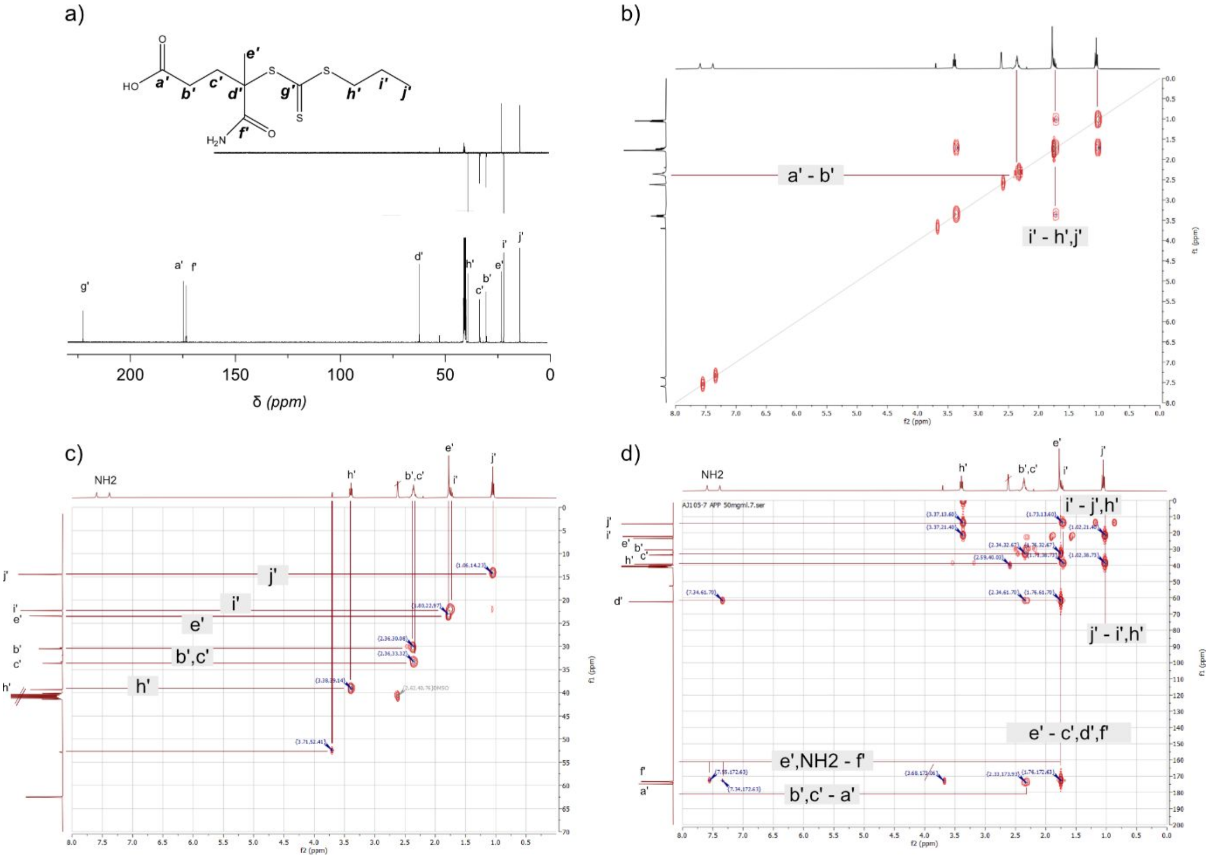

Figure S2: NMR characterization of APP in deuterated DMSO, 50 mg ml^{-1}; (a) ^{13}CNMR and DEPT-135, (b) COSY, (c) HSQC, and (d) HMBC.

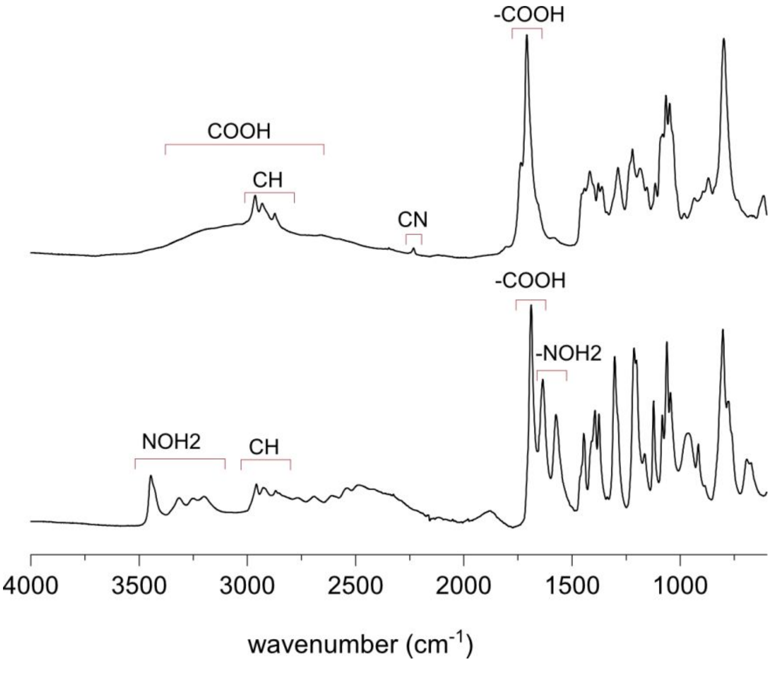

Figure S3: FTIR of CTPPA (upper) and APP (lower). In APP spectrum we clearly see the disappearance of the nitrile signal, and appearance of amide peaks at 3500-3300 and distinct double signal 1635-1575 cm^{-1}.

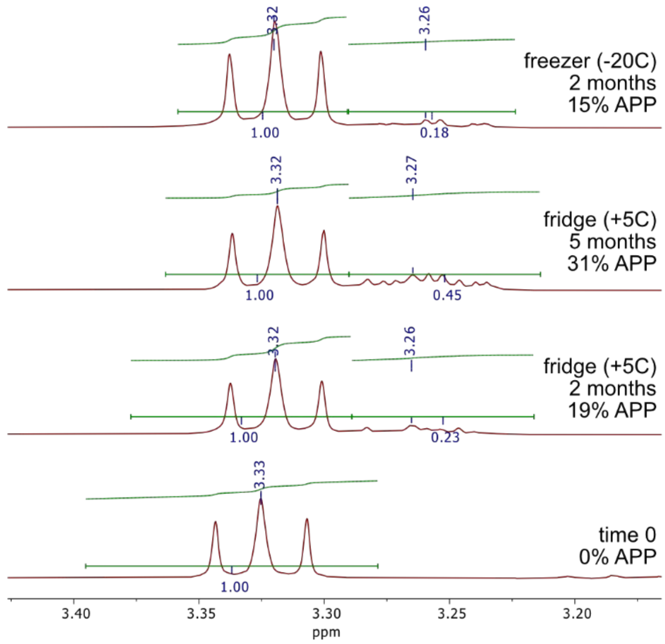

Figure S4: Degradation of CTPPA to APP upon storage under Argon in fridge (+5 ^{o}C) and freezer (-20 ^{o}C). Quantification using CTPPA (S)CH_2 (h) at 3.32 ppm to APP (S)CH_2 (h') at 3.26 ppm.

Kinetics of MMA/THF polymerizations

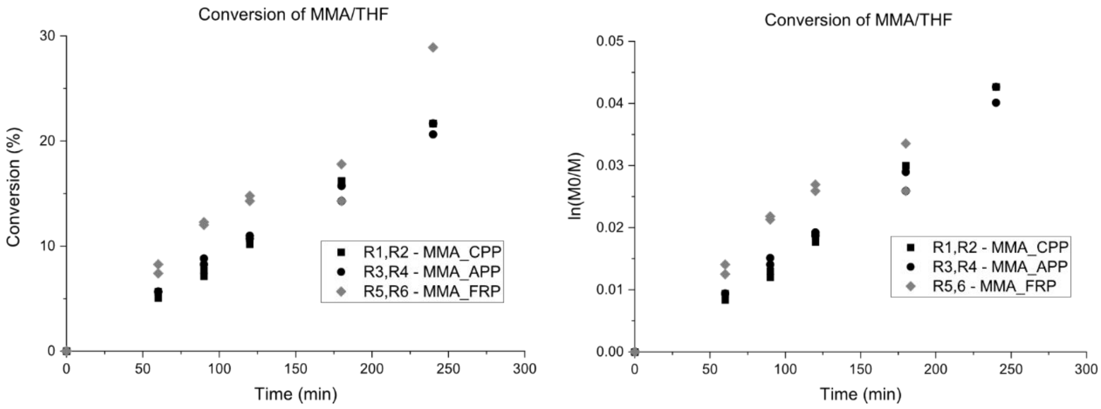

Figure S5: Conversion kinetics of polymerizations of MMA in THF. Conversion calculated from ^{1}HNMR in deuterated chloroform.

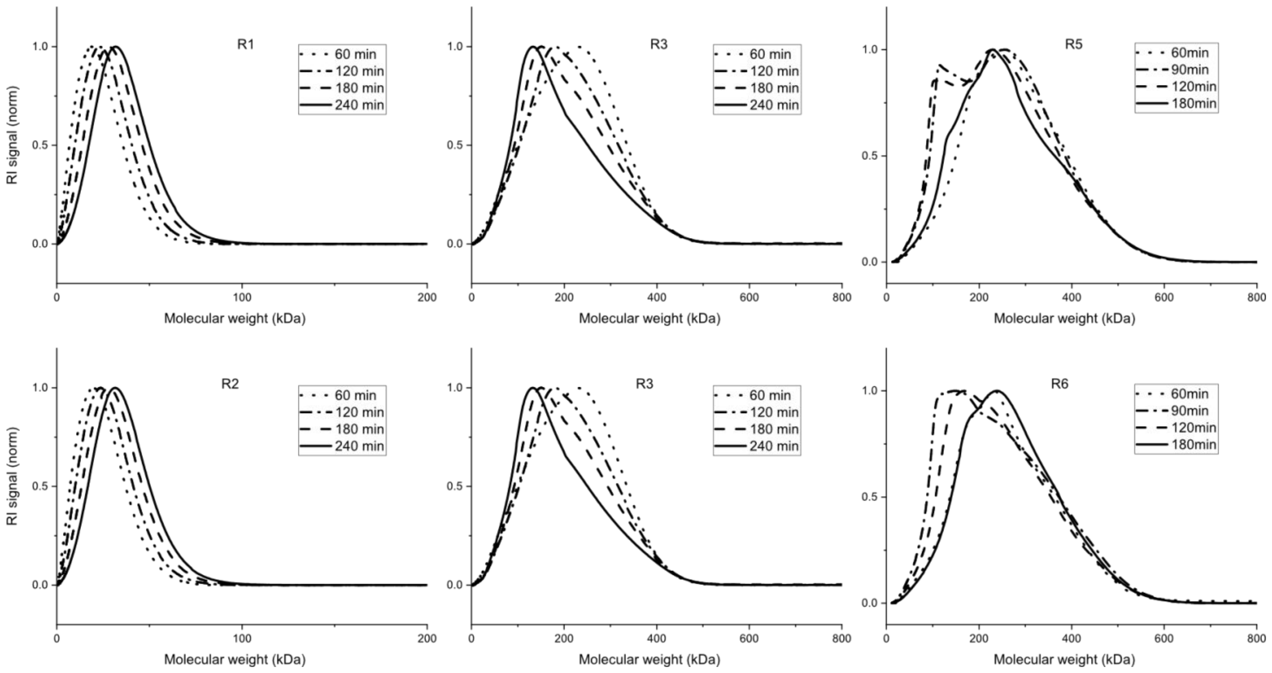

Figure S6: Elugrams showing chain growth in polymerizations of MMA in THF. (left – R1,R2) polymerizations with CTPPA, (middle – R3,R4) polymerizations with APP, and (right – R5,R6) polymerizations without chain-transfer agent, ie free radical polymerization.

Kinetics of DMAEMA/dioxane polymerizations

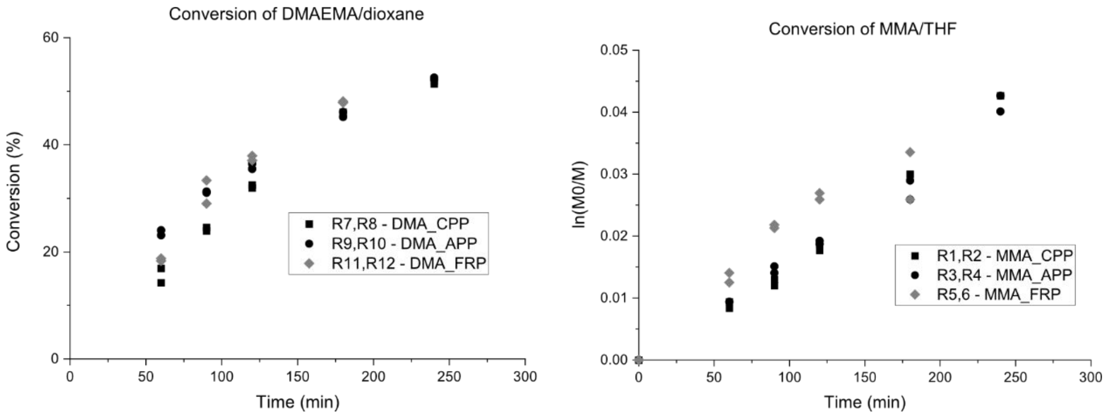

Figure S7: Conversion kinetics of polymerizations of DMAEMA in dioxane. Conversion calculated from ^{1}HNMR in D_2O.

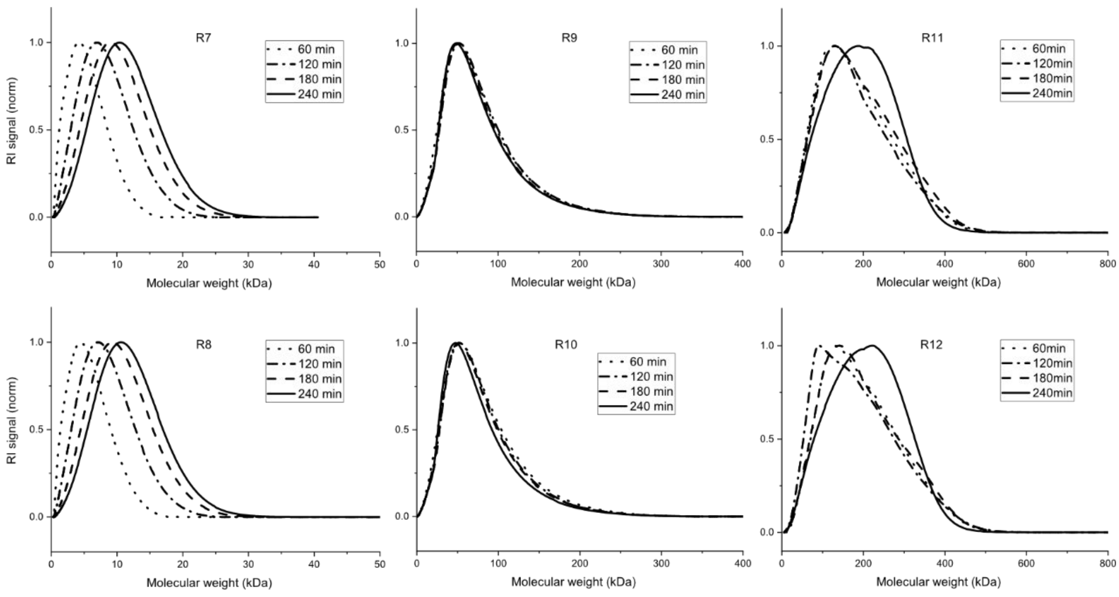

Figure S8: Elugrams showing chain growth in polymerizations of DMAEMA in dioxane. (left – R7,R8) polymerizations with CTPPA, (middle – R9,R10) polymerizations with APP, and (right – R11,R12) polymerizations without chain-transfer agent, ie free radical polymerization.

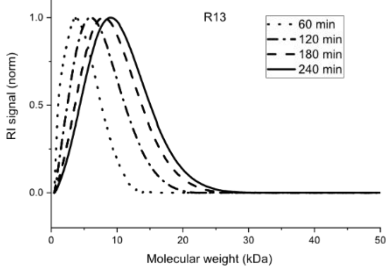

Figure S9: Elugrams showing chain growth in polymerizations of DMAEMA in dioxane with CTPPA and APP 1:1 mol ratio (R13).

Chain extension of PDMAEMA

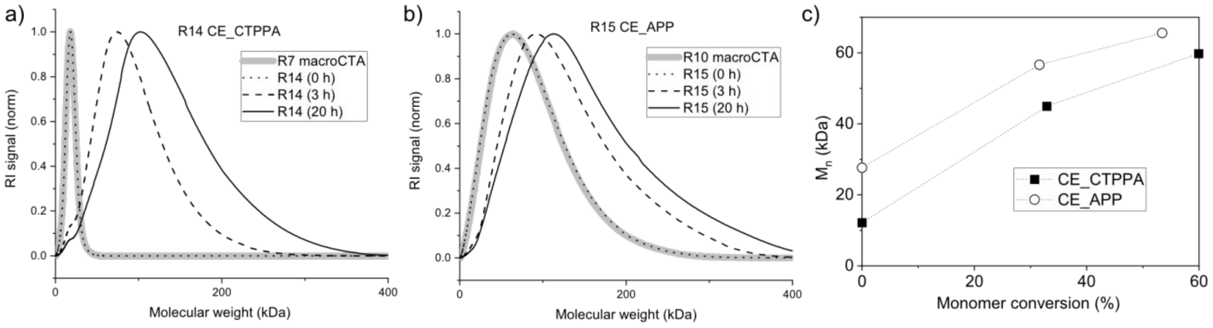

Figure S10: Elugrams showing chain growth in chain extension experiments of PDMAEMA macroCTAs (a) DMA_CTPPA (R7) and (b) DMA_APP (R10) with DMAEMA monomer. (c) Molecular weight evolution with conversion during chain extension.
